# Ventilation distribution measured with electrical impedance tomography at varying levels of assist during pressure support versus neurally adjusted ventilatory assist in ICU patients

**DOI:** 10.1186/cc12078

**Published:** 2013-03-19

**Authors:** MS Van Mourik, P Blankman, D Hasan, D Gommers

**Affiliations:** 1 Erasmus Medical Center, Rotterdam, the Netherlands; 2 Maasstad, Rotterdam, the Netherlands

## Introduction

Electrical impedance tomography (EIT) is a non-invasive and nonradiating imaging technique, which can be used to visualize ventilation distribution of the lungs and could distinguish between the dependent (dorsal) and nondependent (ventral) parts.

## Methods

The aim of this study was to observe ventilation distribution between dependent and nondependent lung regions, for the individual patient, during three different levels of support during pressure support (PS) and neurally adjusted ventilatory assist (NAVA) ventilation. Ten mechanically ventilated patients in the ICU were included.

## Results

The ratio for dependent/nondependent distribution of ventilation is significantly higher at lower support levels compared with higher support levels in both PS and NAVA. However, during NAVA there was significantly less impedance loss between the different levels of assist compared with PS. Tidal volumes decreased when decreasing assist levels during PS whereas not during NAVA ventilation. The electrical activity of the diaphragm decreased in both PS and NAVA with higher levels of assist. Three patients showed an increase in dependent tidal impedance variation (TIV) after lowering the assist level from 15 to 10 cmH_2_O. This increase in TIV did not occur during NAVA ventilation.

## Conclusion

There is more ventilation in the dependent part of the lung, compared with the nondependent part, at lower levels of assist. This could indicate that at higher support levels the contribution of the diaphragm is reduced. During NAVA ventilation, there is an autoregulation in which the patient is adjusting his tidal ventilation to maintain homogeneous ventilation distribution.

